# Honors to Veterinary Educators

**Published:** 1895-06

**Authors:** 


### HONORS TO VETERINARIANS.

The French Government has just conferred the rank of Officer of Agricultural Merit upon Messrs. Chauveau, Member of the Academy of Science, Inspector-General of Veterinary Schools and Officer of the Legion of Honor; Trasbeau, Member of the Academy of Medicine, Director of the National Veterinary School at Alfort, and Officer of the Legion of Honor; Nocard, Member of the Academy of Medicine, Professor at the National Veterinary School at Alfort, and Officer of the Legion of Honor.

The rank of Chevalier of the Legion of Honor has been conferred upon Mr. Alfred Faure, Professor of Natural History at the Veterinary School of Lyons.

